# Post-chemotherapy pneumonia in Chinese patients with diffuse large B-cell lymphoma: Outcomes and predictive model

**DOI:** 10.3389/fonc.2022.955535

**Published:** 2022-08-17

**Authors:** Jinrong Zhao, Yan Zhang, Wei Wang, Wei Zhang, Daobin Zhou

**Affiliations:** Department of Hematology, Peking Union Medical College Hospital, Peking Union Medical College, Chinese Academy of Medical Sciences, Beijing, China

**Keywords:** pneumonia, diffuse large B-cell lymphoma (DLBCL), prognosis, ECOG score, hypoalbuminemia, chemotherapy

## Abstract

Pulmonary infections account for a large proportion of life-threatening adverse events that occur after chemotherapy in patients with diffuse large B-cell lymphoma (DLBCL); however, data on their influencing risk factors and the effects of infection are relatively limited. A total of 605 patients with DLBCL were newly diagnosed at our institution between March 2009 and April 2017, and 132 of these patients developed pneumonia after treatment (21.8%). There was a significant difference in overall survival (OS) between the pneumonia and non-pneumonia groups (hazard ratio 4.819, 95% confidence interval: 3.109–7.470, *p* < 0.0001), with 5-year OS of 41% and 82%, respectively. Pulmonary involvement, Eastern Cooperative Oncology Group score > 1, and hypoalbuminemia were identified as independent risk factors for the development of pneumonia. We constructed a prediction model based on these three factors, and the area under the curve was 0.7083, indicating good discrimination. This model may help clinicians develop individualized strategies for preventing and treating post-chemotherapy pneumonia in patients with DLBCL.

## Introduction

Diffuse large B-cell lymphoma (DLBCL) is the most common subtype of non-Hodgkin’s lymphoma (NHL), accounting for approximately 38% of all NHL cases in China (1). While the R-CHOP regimen, which consists of rituximab, cyclophosphamide, doxorubicin, vincristine, and prednisone, has significantly improved the prognosis of DLBCL patients over the last 20 years, the proliferation of new therapies such as programmed cell death-1 monoclonal antibodies, antibody-conjugated drugs such as Pola, and chimeric antigen receptor-T cell therapy, have also brought hope to patients with recurrent or refractory disease (2). However, the toxicity of chemotherapeutic agents also imposes a major burden on patients, especially in terms of pulmonary infections resulting from chemotherapy. In patients with lymphoma, acute respiratory failure is often the main cause of intensive care unit admission and is associated with shorter survival (3). There are relatively limited data on the risk factors and exposure to infection in DLBCL patients undergoing chemotherapy and identifying high-risk patient groups may help in the targeted application of prophylactic therapy. The aim of the present study was to determine the incidence and risk factors for pneumonia in DLBCL patients undergoing chemotherapy, develop a predictive model for pneumonia, and investigate the relationship between pneumonia and overall survival (OS).

## Patients and methods

### Patients

The clinical data of patients with newly diagnosed DLBCL at our hematology department between March 2009 and April 2017 were analyzed retrospectively. Patients with a history of chest radiotherapy, incomplete clinical data, or who were diagnosed at our hospital but did not undergo follow-up treatment (n = 55) were excluded. Finally, 605 patients were included in the study. Pathological samples from all patients were analyzed independently by two experienced pathologists and diagnosed based on World Health Organization classification criteria for hematopoietic and lymphoid tissue tumors (4). Diagnosis of pulmonary infection requires respiratory symptoms and physical examination in conjunction with radiographic evidence of infiltrates (5). Clinical data collected included sex, age, comorbidities (cardiovascular disease, respiratory disease, chronic kidney disease, diabetes mellitus, hepatitis B), history of smoking, extranodal involvement, chemotherapy regimen, absolute lymphocyte count (ALC), absolute monocyte count (AMC), pre-chemotherapy albumin (ALB), post-chemotherapy level of myelosuppression, rate of pneumonia, rate of severe pneumonia, and microbiological culture results. The study was approved by the Institutional Review Board of Peking Union Medical College Hospital, Chinese Academy of Medical Sciences and conducted in accordance with the Declaration of Helsinki. Written informed consent was exempted because the identity of patients was anonymized.

### Follow-up

Follow-up was conducted *via* telephone, review of patient records, and the electronic follow-up system at our hematology department for lymphoma patients. The cut-off date for follow-up was May 1, 2022. The median follow-up period was 65.1 months (range: 0.1–159.3 months). OS was defined as the time from the date of diagnosis to death from any cause or to the last follow-up visit.

### Construction of prognostic model

Univariate logistic regression analysis was performed after determining the risk factors for pneumonia. Factors with *p* < 0.1 in the univariate analysis were selected for inclusion in the multivariate logistic regression analysis. The risk scoring model was developed based on a risk prediction methodology designed with nested case-controls (6). The risk score was determined by the number of significant risk factors in the multivariate analysis. Point values were assigned to these risk factors based on the ratio of regression coefficients between the factors (7). The performance of the predictive models was assessed using receiver operating characteristic (ROC) curves.

### Statistics

Between-group categorical data were compared using the chi-square test or Fisher’s exact probability test. Survival analysis was performed using the Kaplan-Meier method. The log-rank test was used for comparison between groups. Univariate and multivariate analyses were performed using logistic regression analysis. Data were analyzed using GraphPad 9.0 (GraphPad, San Diego, CA, USA) and SPSS 22.0 statistical software (IBM Corp., Armonk, NY, USA). Differences with *p* < 0.05 were considered statistically significant.

## Results

### Clinical characteristics of patients

Of the patients included in the study between April 2009 and April 2017, 303 (50.1%) were male, and the median age was 58 years (range: 15–90 years). More patients who developed pneumonia were in advanced stages (81.8% vs. 37.2%, *p* = 0.001) and had pulmonary involvement (12.1% vs. 4.2%, *p* = 0.001), B symptoms (65.1% vs. 45%, *p* < 0.001), comorbid chronic respiratory disease (12.1% vs. 5.1%, *p* = 0.004), hypoalbuminemia (56.8% vs. 27.1%, *p* < 0.001), low lymphocytes/monocytes ratio (LMR) (49.2% vs. 36.6%, *p* = 0.008), high lactate dehydrogenase (58.3% vs. 39.1%, *p* < 0.001), poorer physical condition (74.2 vs. 44.4%, *p* < 0.001), and a higher proportion of granulocytopenia (81.8% vs. 25.6%, *p* < 0.001). Specific clinical characteristics are detailed in **Table 1**.

**Table 1:** Clinical characteristics and treatment selection for patients.

Clinical characteristic or treatment selection	Developed pneumonia		
	Yes, n = 132	No, n = 473	p^*^-value
Sex			
male	66 (50%)	237 (50.1%)	0.983
female	66 (50%)	236 (49.9%)	
Age			
≤ 60 years	74 (56.1%)	270 (57.1%)	0.834
> 60 years	58 (43.9%)	203 (42.9%)	
History of smoking			
Yes	27 (20.4%)	116 (24.5%)	0.330
No	105 (79.6%)	357 (75.5%)	
Hans classification			0.616
GCB	68(51.5%)	232(49.0%)	
Non-GCB	64(48.5%)	241(51.0%)	
Ann Arbor stage			
Stages I-II	24 (18.2%)	155 (32.8%)	0.001
Stages III-IV	108 (81.8%)	318 (67.2%)	
Bone marrow involvement			
Yes	32 (24.2%)	39 (8.2%)	<0.001
No	100 (75.8%)	434 (91.8%)	
Lung involvement			
Yes	16 (12.1%)	20 (4.2%)	0.001
No	116 (87.9%)	453 (95.8%)	
ECOG score			
0-1	34 (25.8%)	263 (55.6%)	<0.001
2-4	98 (74.2%)	210 (44.4%)	
B symptoms			
Yes	86 (65.1%)	213 (45%)	<0.001
No	46 (34.9%)	260 (55%)	
Comorbidities			
Diabetes	14 (10.6%)	61 (12.9%)	0.480
Chronic kidney disease	6 (4.5%)	9 (1.9%)	0.084
Cardiovascular disease	45 (34%)	131 (27.7%)	0.153
Chronic respiratory disease	16 (12.1%)	24 (5.1%)	0.004
Chronic hepatitis B infection	14 (10.6%)	36 (7.6%)	0.269
Laboratory data			
ALB			
≤ 35	75 (56.8%)	128 (27.1%)	<0.001
> 35	57 (43.2%)	345 (72.9%)	
LMR			
< 2.7	65 (49.2%)	173 (36.6%)	0.008
≥ 2.7	67 (50.8%)	300 (63.4%)	
LDH			
> 250	77 (58.3%)	185 (39.1%)	<0.001
≤ 250	55 (41.7%)	288 (60.9%)	
Treatment regimen			
R-CHOP	61 (46.2%)	288 (60.9%)	<0.001
R-CHOP+MTX	24 (18.1%)	78 (16.5%)	
R-EPOCH	2 (1.5%)	24 (5.1%)	
Other	45 (34.2%)	83 (17.5%)	
Courses of treatment			
≤4	38 (28.8%)	58 (12.3%)	<0.001
4-8	55 (41.7%)	337 (71.2%)	
>8	39 (29.5%)	78 (16.5%)	
Post-treatment granulocytopenia			
Yes	108 (81.8%)	121 (25.6%)	<0.001
No	24 (18.2%)	352 (74.4%)	

LMR, lymphocyte-to-monocyte ratio; ALB, albumin; LDH, lactate dehydrogenase; GCB, germinal center B-cell;^*^ p-value is calculated using the chi-square test or Fisher’s exact test, differences with p < 0.05 are considered statistically significant.

### Pathology data

Among 163 total cases of pneumonia, severe pneumonia accounted for 23.9% (39/163), and 22.7% (30/132) of patients died from severe infections. The source of infection was confirmed by microbiological culture in 23.5% (31/132) of cases, and the most common pathogens were Gram-negative bacteria, with *Pseudomonas aeruginosa*, *Acinetobacter baumannii*, and *Klebsiella pneumoniae* accounting for 20.7%, 12.7%, and 12.7% of cases, respectively. The specific microorganisms involved are detailed in **Table 2**.

**Table 2:** Microbiologically confirmed infections.

Type of microorganism	Specific microorganism	Number, n = 58
Gram-positive bacteria	*Staphylococcus aureus*	1 (1.8%)
	*Streptococcus haemolyticus*	1 (1.8%)
	*Enterococcus faecalis*	1 (1.8%)
	*Streptococcus pneumoniae*	1 (1.8%)
Gram-negative bacteria	*Escherichia coli*	3 (5.2%)
	*Acinetobacter baumannii*	7 (12.7%)
	*Stenotrophomonas maltophilia*	3 (5.2%)
	*Klebsiella pneumoniae*	7 (12.7%)
	*Bacteroides fundili*	1 (1.8%)
	*Pseudomonas aeruginosa*	12 (20.7%)
	*Enterobacter cloacae*	1 (1.8%)
	*Burkholderia cepacia*	1 (1.8%)
Fungi	*Candida* spp.	10 (17.2%)
	*Aspergillus* spp. *Pneumocystis carinii*	7 (12.7%) 1 (1.7%)
Viruses	Cytomegalovirus	1 (1.7%)

### Survival

**Figure 1:**
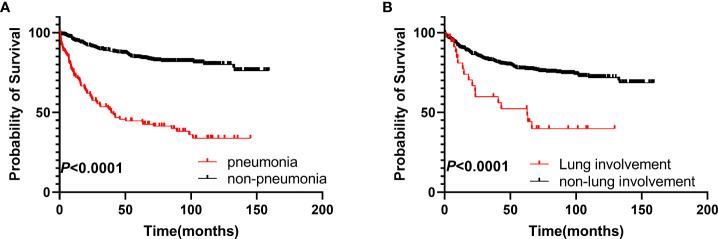
Survival analysis of the two groups. **(A)** OS of patients in the pneumonia group compared to those in the non-pneumonia group. **(B)** OS of patients with pulmonary involvement compared to those without pulmonary involvement. OS, overall survival.

Patients who developed pneumonia had poorer OS compared to the non-pneumonia group (hazard ratio [HR] 4.819, 95% confidence interval [CI]: 3.109–7.470, *p* < 0.0001), with 5-year OS rates of 41% and 82%, respectively (**Figure 1A**). Patients with lung involvement also had poorer survival (HR 2.750, 95% CI: 1.265–5.979, *p* < 0.0001), with 5-year OS rates of 39% and 76%, respectively (**Figure 1B**).

### Risk factors for developing pneumonia

The results of univariate and multifactorial analyses for development of pneumonia (**Table 3**) show that progressive stage, bone marrow involvement, lung involvement, > 1 site of extranodal involvement, Eastern Cooperative Oncology Group (ECOG) score > 1, B symptoms, large masses, chronic respiratory disease, low ALB, and low LMR were significantly associated with development of pneumonia. However, multifactorial analysis showed that lung involvement, ECOG score > 1, and low ALB were independent risk factors for development of pneumonia.

**Table 3:** Risk factors for development of pneumonia in DLBCL patients.

Variable	Univariate		Multivariate	
	p^*^-value	OR	95%	p^*^-value
Male sex	0.983			
Age > 60 years	0.834			
History of smoking	0.330			
Progressive stage	0.001			
Bone marrow involvement	<0.001			
Extranodal involvement sites > 1	0.006			
Lung involvement	0.001	3.242	1.375–7.642	0.007
ECOG score > 1	0.001	0.477	0.285–0.798	0.005
B symptoms	<0.001			
Diabetes	0.480			
Chronic kidney disease	0.084			
Cardiovascular disease	0.153			
Chronic respiratory disease	0.004			
Chronic hepatitis B infection	0.269			
ALB≤ 35	<0.001	2.006	1.229–3.274	0.005
Low LMR	0.008			

LMR, lymphocyte-to-monocyte ratio; ALB, albumin; ECOG, Eastern Cooperative Oncology Group.

^*^p-value calculated from logistic regression analysis, differences with p < 0.05 considered statistically significant.

### Prediction model for pneumonia

**Figure 2:**
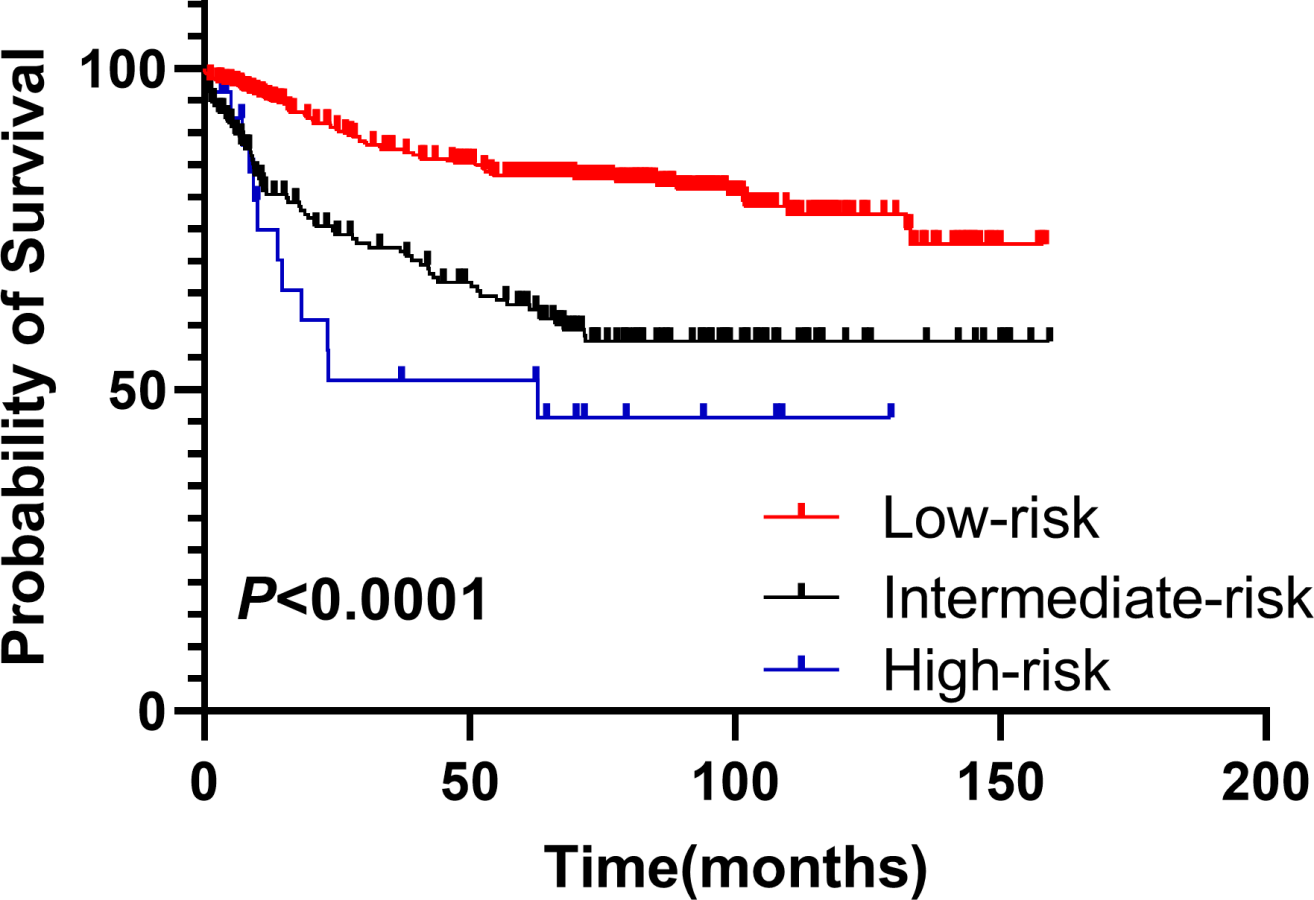
Stratification of OS of DLBCL patients using the prediction model. Overall survival of patients in groups classified as low-, intermediate -, and high-risk based on the pneumonia prediction model. DLBCL, diffuse large B-cell lymphoma; OS, overall survival.

**Figure 3:**
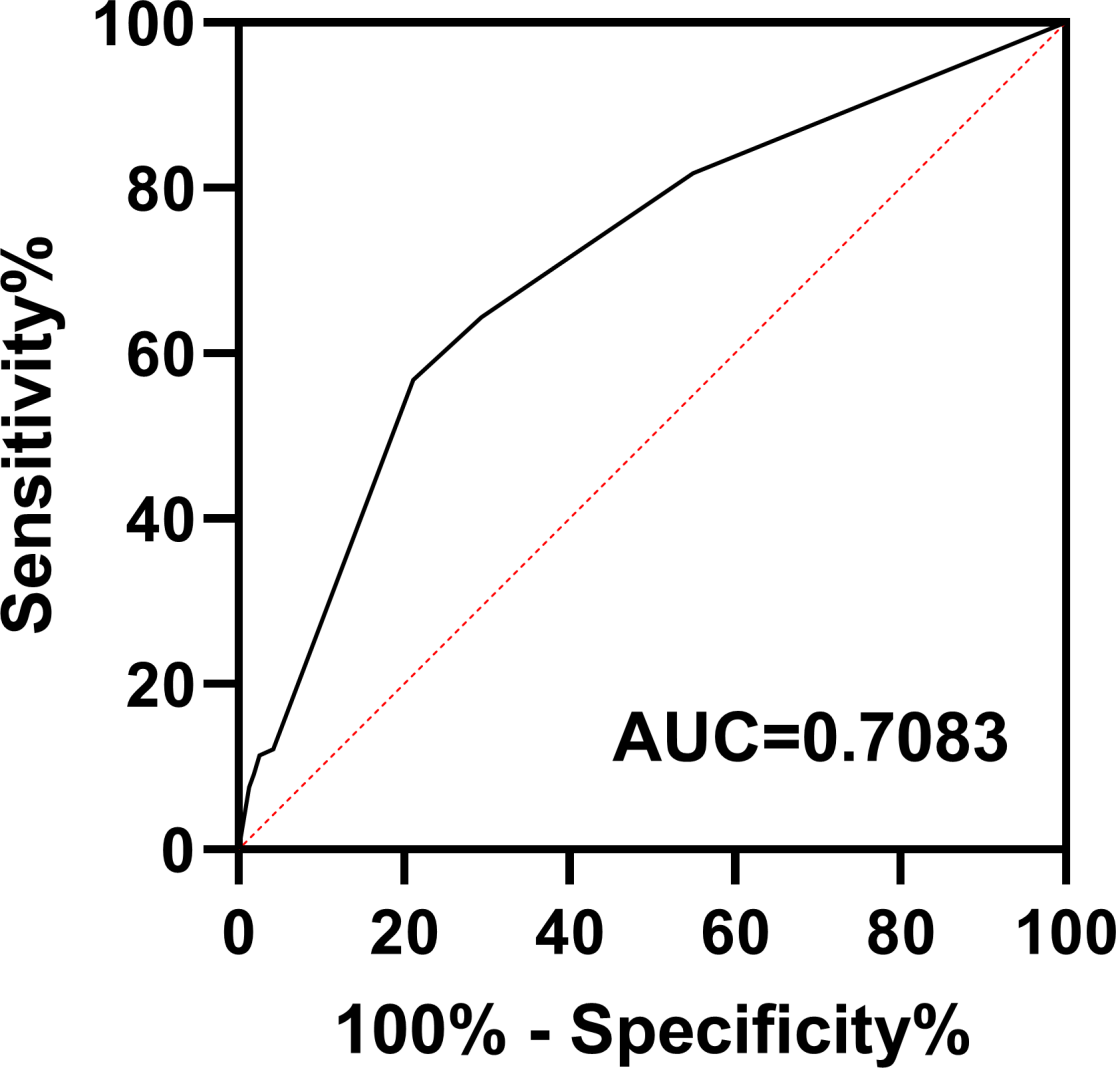
Receiver operating characteristic curve for the pneumonia prediction model. AUC, area under the curve.

Based on the results of the multifactorial analysis, we constructed a prediction model for development of pneumonia in patients with DLBCL (**Table 4**). Each patient was assigned a score (0–12 points) according to the model and then classified into one of three groups: low risk (0–1 points), moderate risk (4–7 points), and high risk (8–12 points). The probability of pneumonia in each risk subgroup was 13.6% (53/387), 33.5% (64/191), and 55.5% (15/27), respectively. The mortality rate for patients was 17.3% (67/387) in the low-risk group, 35.6% (68/191) in the moderate-risk group, and 44.4% (12/27) in the high-risk group (*p* < 0.0001, **Figure 2**). Our prediction model had good discriminatory power (area under the curve [AUC] = 0.7083, 95% CI: 0.6572–0.7595, *p* < 0.0001) (**Figure 3**).

**Table 4:** Prediction model for pneumonia.

Variable	*p*-value	OR (95%)	Regression coefficient	Score
Lung involvement	0.007	3.242 (1.375–7.642)	1.379	7
ECOG score > 1	0.005	0.477 (0.285–0.798)	0.211	1
ALB≤ 35	0.005	2.006 (1.229–3.274)	0.816	4

ALB, albumin; ECOG, Eastern Cooperative Oncology Group.

## Discussion

Previous studies have shown that the use of rituximab significantly improves survival in patients with DLBCL without increasing the incidence of adverse events such as infections (8, 9). However, development of pulmonary infections in patients with hematologic neoplasms is often fatal, and infection is inherently an independent predictor of death in patients with DLBCL (10). With the growing number of patients with lymphoma, the need for systematic investigation of post-immunochemotherapy pneumonia in DLBCL patients is ever increasing.

Compared with 5.6–29.3% reported in previous studies (10–13), in the present study, 21.8% of patients developed pneumonia while undergoing treatment. In newly diagnosed DLBCL patients, chronic underlying respiratory disease, poor physical condition, high tumor burden, and multiple extranodal organ involvement tend to increase susceptibility to pulmonary infection (14). Unexpectedly, the rate of pulmonary infection was not significantly higher in elderly patients, which may be associated with lower doses of chemotherapy. In addition, absolute lymphocyte count (ALC) is considered a good proxy for immune status, whereas LMR is a good indicator of immune function and tumor microenvironment crosstalk (15). Our data also suggest that patients who develop pneumonia tend to have a lower LMR. Hypoalbuminemia can lead to decreased pulmonary osmolarity, which leads to increased extravascular lung water and exacerbation of hypoxemia, thereby increasing the risk of pulmonary infection (16–18). Differences in treatment regimen and number of treatment cycles also have major impacts on outcomes (19). In patients in the pneumonia group, the rate of severe myelosuppression was significantly higher after treatment. Granulocytopenia is a strong predictor of infectious episodes, and the use of granulocyte colony-stimulating factor (G-CSF) reduces the risk of severe granulocytopenia and its associated infections (20). Lung involvement in patients with lymphoma increases respiratory complications (21, 22), and our data confirm a significantly higher incidence of pneumonia in patients with lung involvement, which is associated with a poor prognosis.

According to studies, infectious episodes, especially pulmonary infections, are associated with poor prognosis in patients with DLBCL (10). Data from our institution showed that patients who had suffered from pulmonary infections had half the 5-year OS rate compared to other patients, and 22.7% of patients with pneumonia died due to severe infection or respiratory failure. It is increasingly important to identify these patients who are at high risk of developing pneumonia so that targeted treatment, such as adjustment of chemotherapy drug regimen and dose and post-treatment support measures such as G-CSF, can be administered. In the present study, we developed a prediction model for development of pneumonia in patients with DLBCL after treatment, which incorporated three relatively easily available indicators: lung involvement, hypoalbuminemia, and physical condition. The incidence of pneumonia and clinical outcomes of DLBCL patients in each risk group differentiated according to the model were significantly different, and the AUC values were relatively high, indicating that the model has good predictive power. With respect to strategies for preventing infection in high-risk patients, good patient education and increased surveillance frequency are recommended, but prophylactic use of antimicrobial drugs after chemotherapy remains controversial. Previous studies have shown that prophylactic use of antimicrobial drugs such as levofloxacin in patients with lymphoma has a positive effect on patients (23, 24). Thus, clinicians are recommended to give more consideration to prophylactic antibiotic therapy in high-risk patients.

The primary limitation of this study was that it is a single-center retrospective study. In addition, patients are often out-of-hospital when they develop infections, which can lead to some missing data, and the number of infectious episodes and microbial culture results were not available in a timely manner. The model we developed also must be validated with a larger sample and an external study cohort.

In conclusion, our study demonstrates that pneumonia is a relatively common complication after treatment in patients with DLBCL, and it negatively affects patient prognosis. Independent risk factors for pneumonia were identified, and a predictive model for the development of pneumonia was constructed, which may help clinicians develop individualized pneumonia prevention and treatment strategies.

## Data availability statement

The original contributions presented in the study are included in the article/supplementary material. Further inquiries can be directed to the corresponding author.

## Ethics statement

The studies involving human participants were reviewed and approved by Institutional Review Board of Peking Union Medical College Hospital, Chinese Academy of Medical Sciences. Written informed consent for participation was not required for this study in accordance with the national legislation and the institutional requirements.

## Author contributions

JZ analyzed and interpreted the patient data and was a major contributor in writing the manuscript. DZ and WZ conceived and designed the work that led to the submission, acquired data and had an important role in interpreting the results. YZ and WW had contributed to data collection and patient management. WZ gave final approval of the version to be published. All authors read and approved the final manuscript.

## Funding

This work was supported by the National Natural Science Foundation of China (No.81970188).

## Acknowledgments

We would like to thank Editage (www.editage.cn) for English language editing. Thanks to all the patients and their families, thanks to Dr. Sun Xueyan for her help in statistics.

## Conflict of interest

The authors declare that the research was conducted in the absence of any commercial or financial relationships that could be construed as a potential conflict of interest.

## Publisher’s note

All claims expressed in this article are solely those of the authors and do not necessarily represent those of their affiliated organizations, or those of the publisher, the editors and the reviewers. Any product that may be evaluated in this article, or claim that may be made by its manufacturer, is not guaranteed or endorsed by the publisher.
